# Transitional care from skilled nursing facilities to home: study protocol for a stepped wedge cluster randomized trial

**DOI:** 10.1186/s13063-021-05068-0

**Published:** 2021-02-05

**Authors:** M. Toles, C. Colón-Emeric, L. C. Hanson, M. Naylor, M. Weinberger, J. Covington, J. S. Preisser

**Affiliations:** 1 School of Nursing, University of North Carolina at Chapel Hill, Chapel Hill, USA; 2 School of Medicine and the Durham VA GRECC, Duke University, Durham, USA; 3 School of Medicine, University of North Carolina at Chapel Hill, Chapel Hill, USA; 4 School of Nursing, University of Pennsylvania, Philadelphia, USA; 5 Gillings School of Global Public Health, University of North Carolina at Chapel Hill, Chapel Hill, USA

**Keywords:** Transitional care, Skilled nursing facilities, Patient discharge, Caregivers, Aging, Frailty

## Abstract

**Background:**

Skilled nursing facility (SNF) patients are medically complex with multiple, advanced chronic conditions. They are dependent on caregivers and have experienced recent acute illnesses. Among SNF patients, the rate of mortality or acute care use is over 50% within 90 days of discharge, yet these patients and their caregivers often do not receive the quality of transitional care that prepares them to manage serious illnesses at home.

**Methods:**

The study will test the efficacy of Connect-Home, a successfully piloted transitional care intervention targeting seriously ill SNF patients discharged to home and their caregivers. The study setting will be SNFs in North Carolina, USA, and, following discharge, in patients’ home. Using a stepped wedge cluster randomized trial design, six SNFs will transition at randomly assigned intervals from standard discharge planning to the Connect-Home intervention. The SNFs will contribute data for patients (*N* = 360) and their caregivers (*N* = 360), during both the standard discharge planning and Connect-Home time periods. Connect-Home is a two-step intervention: (a) SNF staff create an individualized Transition Plan of Care to manage the patient’s illness at home; and (b) a Connect-Home Activation RN visits the patient’s home to implement the written Transition Plan of Care. A key feature of the trial includes training of the SNF and Home Care Agency staff to complete the transition plan rather than using study interventionists. The primary outcomes will be patient preparedness for discharge and caregiver preparedness for caregiving role. With the proposed sample and using a two-sided test at the 5% significance level, we have 80% power to detect a 18% increase in the patient’s preparedness for discharge score. We will employ linear mixed models to compare observations between intervention and usual care periods to assess primary outcomes. Secondary outcomes include (a) patients’ quality of life, functional status, and days of acute care use and (b) caregivers’ burden and distress.

**Discussion:**

Study results will determine the efficacy of an intervention using existing clinical staff to (a) improve transitional care for seriously ill SNF patients and their caregivers, (b) prevent avoidable days of acute care use in a population with persistent risks from chronic conditions, and (c) advance the science of transitional care within end-of-life and palliative care trajectories of SNF patients and their caregivers. While this study protocol was being implemented, the COVID-19 pandemic occurred and this protocol was revised to mitigate COVID-related risks of patients, their caregivers, SNF staff, and the study team. Thus, this paper includes additional material describing these modifications.

**Trial registration:**

ClinicalTrials.gov NCT03810534. Registered on January 18, 2019.

## Introduction

### Background and rationale

Annually in the USA, 1.6 million older adults undergo episodes of care consisting of hospitalization followed by a “short stay” in a skilled nursing facility (SNF) for rehabilitation, medical and nursing care [1]. After returning home, SNF patients are at high risk for return to acute care, continued functional decline, and death [2–5]. In our analysis of 55,000 SNF patient transitions to home, within 90 days of being discharged home from the SNF, 25.9% used emergency department (ED) services, 20.1% were rehospitalized, and 8.1% died [6].

The morbidity and frailty of SNF patients increases the risk of poor outcomes after transitions from SNFs to home. More than half of patients are greater than 80 years old, and nearly all have recent acute illness (e.g., hip fracture, stroke, genitourinary, and pulmonary infections with or without sepsis) [6–8]. Most SNF patients also have underlying, incurable chronic conditions, such as congestive heart failure, chronic obstructive pulmonary disease, and diabetes. About a third are cognitively impaired [6, 7, 9] and most experience multimorbidity. SNF patients also have a high rate of geriatric syndromes (e.g., depressive symptoms, poor sleep, unplanned weight loss) and frequently require routine assistance from family and friends for activities of daily living [10, 11]. To successfully transition from short stays in SNFs to home-based care, seriously ill SNF patients and their caregivers require a range of community-based palliative care supports, such help prioritizing what matters most to patients and families, plans for symptom management, coordination of care, and additional homecare or assistive devices to compensate for functional losses [12, 13].

A potential limitation in community-based care of adults with serious illness is the quality of discharge planning in SNFs [14–16]. Findings from our case studies of transitional care indicate that SNF staff frequently lacked awareness of the key care needs of SNF patients at home [17, 18]; for example, the need for guidance to prevent falls and to organize visits with follow-up physicians [17, 18]. SNF staff also lacked resources to coordinate discharge planning, such as tools in electronic health records (EHR) systems and a standard schedule for assessing and planning discharge needs with spouses, children, and friends of SNF patients (caregivers) [18]. Finally, SNF staff did not consistently teach caregivers skills for administering medications, helping with home-based rehabilitation, and managing symptoms of serious illness, such as infections and challenges related to dementia [19–21]. Thus, consistent with evidence from studies of hospitalized patients, improving outcomes for SNF patients and their caregivers likely requires a change in treatment approach—from usual discharge planning to evidence-based transitional care [22–27].

Transitional care is a set of time-limited services designed to promote continuity and coordination of care of frail older adults and their caregivers during transfers between providers and settings of care [24, 28]. Evidence from hospital-based studies demonstrates that transitional care improves patients’ preparedness for discharge and decreases the rate of rehospitalization within at least 30 days of discharge from hospital to home [29–31]. In our systematic review of SNF-based transitional care, we identified promising evidence that transitional care prepares patients and caregivers for home-based care and improves SNF patient and caregiver outcomes; however, prior studies have not evaluated the efficacy of SNF-based transitional care; moreover, most hospital-based studies required dedicated study interventionists, and there is a need for solutions using existing infrastructure and staff [32]. Therefore, in collaboration with stakeholders in SNFs and national experts in nursing, geriatric medicine, and transitional care, we designed Connect-Home, a team-based transitional care intervention to prepare SNF patients and caregivers to manage the patient’s serious illness at home.

The pre-discharge elements of the Connect-Home intervention were pilot tested in a non-randomized, historically controlled study [33]. In the pilot test, we enrolled 133 SNF patients and 133 caregivers in 3 SNFs. Using a standardized protocol, we implemented Connect-Home EHR tools and trained existing SNF staff to use a scheduled routine to provide transitional care in two steps: (a) create and implement a Transition Plan of Care and (b) call patients at home to reinforce transition plans within 72 h of discharge [33, 34]. We found that Connect-Home was feasible, as indicated by completion of written transition plans (90%), follow-up appointment scheduling (90%), medical records transfer to next providers (82%), and post-discharge calls (75%). SNF staff reported that Connect-Home was highly acceptable, and 97% recommended its use in the future. We found that Connect-Home improved patients’ preparedness for discharge. Compared to controls, intervention patients reported being more prepared for discharge (Care Transitions Measure-15 scores, 74.7 vs. 65.3, mean ratio 1.16, 95% CI 1.08, 1.24) [33]. In the pilot study, caregivers reported that SNF patients had unmet needs and the caregivers still needed additional support to self-manage the patient’s serious illness at home. Thus, to enhance the potential for Connect-Home to support patients and caregivers at home and to minimize days of acute care use, we added the “Connect-Home Activation Visit”, a home visit with a registered nurse (employed in a home health care agency) within 24 h of discharge. Finally, we held focus groups with home health care nurses to assess their perceptions of the feasibility of the Connect-Home Activation Nurse visit; nurses unanimously endorsed the significance of follow-up home visits and the feasibility of using the Connect-Home protocol in routine clinical practice with SNF patients and their caregivers.

### Objectives

The objective of this study is to test the efficacy of Connect-Home using a stepped wedge cluster randomized trial design, with blinded outcomes assessment, for seriously ill patients discharged to home (*N* = 360) and their caregivers (*N* = 360) in 6 SNFs [35]. The specific aims are:

1. Evaluate the efficacy of Connect-Home to improve SNF patient and caregiver preparedness for care at home. Hypothesis 1a: Compared to patients enrolled in control periods, Connect-Home patients will experience greater preparedness for discharge (primary outcome, measured with the Care Transitions Measure-15 (CTM-15)) [36], assessed 7 days after discharge home. Hypothesis 1b: Compared to caregivers of patients enrolled in control periods, Connect-Home caregivers will experience greater preparedness for caregiving (primary outcome, measured with the Preparedness for Caregiving Scale) [37], assessed 7 days after discharge home.
2. Evaluate the efficacy of Connect-Home to improve SNF patient and caregiver outcomes after discharge home. Hypothesis 2a: Compared to patients enrolled in control periods, Connect-Home patients will experience better quality of life and function (secondary outcomes) and fewer falls requiring medical assistance (exploratory) 30 and 60 days after discharge home. Hypothesis 2b: Compared to caregivers from control periods, Connect-Home caregivers will experience less burden and caregiver distress (secondary outcomes) 30 and 60 days after discharge home.
3. Evaluate the efficacy of Connect-Home to prevent acute care use up to 60 days after SNF discharge. Hypothesis 3a: Compared to patients enrolled in control periods, Connect-Home patients will have fewer days of acute care use (composite of emergency department and hospital use) (secondary outcome) and more hospice enrollment (exploratory) 30 and 60 days after discharge home.

The rationale for the study is that findings from testing the efficacy of Connect-Home will provide evidence that an innovative model to support seriously ill SNF patients and caregivers during high-risk transitions from SNFs to home can improve patient and caregiver outcomes at home.

## Methods

### Study design

**Fig. 1:**
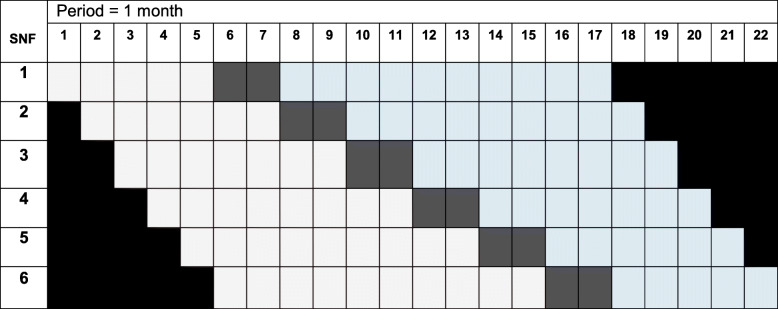
Stepped wedge design with treatment condition by month for 6 SNFs enrolling 4 patients and their caregivers per month (*N* = 360 patients and 360 caregivers)

The study design will be an incomplete, stepped wedge cluster randomized trial to test Connect-Home (intervention) against standard discharge planning (control) for patients discharged to home (*N* = 360) and their caregivers (*N* = 360) in 6 SNFs [35]. In this design, SNFs (clusters) are randomly allocated to six sequences of time-periods (Fig. 1) [38].

The randomized allocation sequence of the SNF determines the beginning and end dates for three phases of patient and caregiver enrollment: (1) standard care (control condition), (2) the pre-implementation phase when staff are trained and neither patients nor caregivers are enrolled, and (3) the intervention phase when newly enrolled patients and their caregivers receive Connect-Home. The beginning of each phase as indicated in Fig. 1 will be on the first Monday of the assigned calendar month in accord with the allocated sequence. Variation in the beginning of the intervention phase was required to accommodate the availability of staff in the SNFs to participate in staff training activities. In this cluster level, unidirectional crossover design, patients, and their caregiver receive either standard discharge planning or Connect-Home consistent with the assigned treatment condition of the SNF at the time of the patient’s enrollment. Due to cost and logistical reasons, the study design is “incomplete” in that data collection does not occur in some cluster periods (black boxes) due to the staggered initiation and termination of SNFs’ trial participation; nor does it occur during staff training periods (green boxes).

*COVID Modification*. At the end of period 12 of this 22-period study (Fig. 1), onset of the COVID pandemic required a pause in all study activities for 6 months. After the 6-month pause, the study was restarted in period 13 exactly as described above.

### Study setting

We will conduct this study in six U.S. SNFs owned by one organization. Inclusion criteria for SNFs are being located within 120 miles of the University of North Carolina at Chapel Hill and having at least 15 post-acute admissions per month. For participants in the intervention periods, Connect-Home Activation RNs, employed by a home health agency owned by the SNF parent organization, will visit the patient and caregiver at home within 24 h of SNF discharge.

### Eligibility criteria

We will recruit 360 SNF patients and 360 caregivers. Inclusion criteria for patients: (a) able to speak English; (b) have a Minimum Data Set 3.0, Section GG Mobility Assessment Score of 3 or less, indicating the patient requires at least 25–50% assistance for functional mobility [39]; (c) be diagnosed with at least one serious medical illness (neurodegenerative dementia, cancer, chronic kidney disease, cirrhosis, congestive heart failure, chronic obstructive or interstitial lung disease, acute infection with sepsis, acute major motor stroke, acute coronary syndrome, acute hip fracture, diabetes with end organ complications, or intensive care for greater than 3 days while hospitalized), and (d) have a caregiver, living with or apart from the patient, and willing to be enrolled in the study. For patients with cognitive impairment, additional criteria are as follows: (a) documentation in the medical record of a caregiver who is the patient’s legally authorized representative; and (b) consent of the caregiver to participate in the study as the patient’s representative. The only exclusion criterion for patients is a planned hospital readmission for procedures/treatments in next 90 days. Inclusion criteria for caregiver/designated responsible party are as follows: self-reports of assisting the patient at home and the ability to speak English.

### Informed consent

**Fig. 2:**
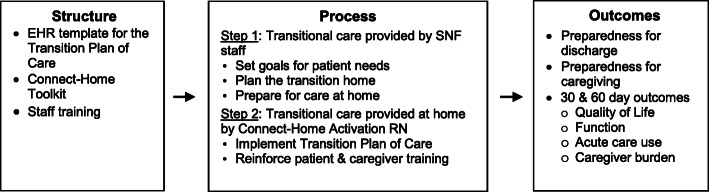
Connect-Home: concepts and key variables

Using a Health Insurance Portability and Accountability Act waiver allowing pre-screening for eligibility, the recruitment coordinator (RC) will (a) consult with a quality control nurse (i.e., the “Minimum Data Set Nurse”) and social worker in each SNF weekly to identify patients expected to discharge from the SNF to home; and (b) screen the medical record of patients expected to discharge home and identify patients meeting inclusion criteria. Using Institutional Review Board (IRB)-approved forms and consent procedures, the RC will sequentially recruit SNF patients and their caregivers within 10 days of admission until the recruitment goal in the SNF for that month is reached (Fig. 2). For patients with cognitive impairment (as indicated by documented diagnosis of dementia and/or a Brief Interview for Mental Status score of greater than 12 in Section C of the Minimum Data Set) [39], the RC will recruit the patient’s legally authorized representative to respond to survey questions as the patient’s proxy [40, 41]. To recruit potential patients and caregivers sequentially, the RC will screen and recruit all patients and caregivers in the order that patients are admitted to the SNF, until the recruitment in the SNF is complete for the study month. Based on study site data, we expect the majority of patients admitted to the study SNFs will be White and female; thus, to ensure that minorities and men are represented in the sample, the RC will make additional outreach efforts to recruit male and Black patients (e.g., allocation of time in the recruitment procedure to facilitate family discussions about patient participation). The RC will recruit patients and caregivers in the SNFs through face-to-face interviews and, if necessary, by telephone for caregivers. All participants will provide written informed consent for study participation; legally authorized representatives will provide consent for patients with cognitive impairment.

*COVID Modification*. At the end of period 12 of this 22-period study (Fig. 1), all study activities were paused. After the pause, risk mitigation strategies were required to minimize the risk of COVID infection and the study team was no longer permitted to enter the SNFs. Thus, the recruitment procedures were revised as follows. First, the study team provided an iPad with Zoom software to staff in each study SNF. Second, the study team trained a staff person in each SNF to support recruitment activities via iPad with Zoom software. Staff were trained to initiate recruitment conversations with patients, using the iPad, to facilitate a recruitment conversation with the patient and a member of the study team. Working remotely, via the Zoom link, a member of the study team explained the purpose of the research to the patient and obtained verbal consent for study participation. All of the revised procedures were IRB approved.

### Study groups

#### Controls

The control comparator will be usual discharge planning services provided for SNF patients, which typically include assignment to an interdisciplinary team who develop discharge instructions for the SNF patients to follow at home with oversight by physicians. In most facilities, a social worker in consultation with the physician and therapist develops a discharge plan with referrals for home care and a discharge medication list, and a nurse provides patient education about the need for medical equipment and schedules follow-up appointments at home [17, 18]. Based on prior research [15, 18, 42], the components of discharge planning in SNFs vary widely, with limited caregiver engagement or outreach to post-discharge providers.

#### Intervention: Connect-Home

Connect-Home transitional care is designed to meet six key care needs of SNF patients and their caregivers: (a) home safety and level of assistance evaluation (a safe discharge destination and a caregiver for assistance at home); (b) advance care planning (surrogate decision makers, prior written advance directives, and preferences regarding resuscitation); (c) symptom management (preparation to manage symptoms of serious illnesses at home, such as pain and dyspnea); (d) medication reconciliation (current medication list and plan for use at home); (e) function and activity training (skill-building for transfers, mobility, and preventing falls); and (f) coordination of care (information relays and follow-up plans with primary care and/or specialist clinicians). Addressing these care needs, Connect-Home provides new organizational supports for SNF staff and home health care RNs to deliver transitional care processes and to build the capacity of patients and caregivers to achieve their healthcare goals at home (Fig. 2) [33].

As described in Table 1, Connect-Home will include new elements of organizational structure for existing SNF staff to provide transitional care for patients discharged home and their caregivers [33, 34, 43].

**Table 1:** Connect-Home: transitional care structure

Structure	Supports for staff delivery of transitional care processes
EHR tool	**Transition Plan of Care Template** Before randomization, an EHR template will be installed in the SNF EHR system. It will contain free text fields in 6 key care domains that SNF staff and home visit RNs will use to record transition goals and deliver the two-step intervention [34].
Toolkit	**Connect-Home Toolkit** All staff will be given 36-page workbook that includes the 2-step intervention protocol (Table 2), checklists and cue sheets, and the intervention schedule.
Staff Training	**Site Leadership Training** (1 h) Principal investigator (PI) will train 1–2 SNF project leaders. Training content: Connect-Home protocol and study procedures. Teaching strategy: Presentation, the Connect-Home Toolkit, and teach-back
	Staff Training (4 h) In each SNF, the PI will train social workers, nurses (RN, LPN, NP), rehabilitation therapists (PT, OT, ST, COTA, PTA) and the Home Health RN. Training content: (a) Patient and caregiver key care needs. (b) Protocols for using the two-step intervention to address key care needs. (c) Using the EHR template to individualize patients’ Transition Plan of Care. (d) Advance care planning. (e) Integrating SNF-based and home-based care (e.g., clinical hand-offs from SNF to Connect-Home Activation RN, home health care, and follow-up medical providers). Teaching strategy: Presentation, review of the Connect-Home Toolkit, and teach-back.
	**Home Health Care RN** (1.5 h). The PI provides additional training for the Connect-Home Activation RN for each SNF (Table 2, below). Training content: Strategies for implementing transition plans of care, home safety screening, responding to medical needs, and handing-off care to home health nurses (when applicable). Teaching strategy: One-to-one instruction and return demonstration.
	**New Staff Training** (1–2 h, as needed): The PI trains replacement staff to use the Connect-Home protocols.

*RN* registered nurse, *LPN* licensed practical nurse, *DON* director of nursing, *MDS* Minimum Data Set Nurse, *NP* nurse practitioner, *PT* physical therapist, *OT* occupational therapist, *ST* speech therapist, *COTA* certified occupational therapy assistant, *PTA* physical therapy assistant

After new structural elements are in place, SNF staff will use Connect-Home transitional care processes to deliver the 2-step transitional care intervention (Table 2). In Step 1, SNF staff will develop a Transition Plan of Care and prepare the patient and caregiver to manage the patient’s serious illness at home. In Step 2, the Connect-Home Activation RN will visit the patient and caregiver at home within 24 h of discharge. The nurse will work with them to activate the Transition Plan of Care at home.

**Table 2:** Connect-Home: transitional care processes (time estimates based on 20-day SNF stay)

Process	Patient/caregiver services and supports	Days
**Step 1.** Transitional care in the SNF	**Set goals for home-based care** (45 min) Consulting with the patient/caregiver, SNF staff use the EHR template to identify goals to address the 6 key care needs of patients and their caregivers. ● Nurses create goals for treatments and responses to symptoms or other health changes ● Rehabilitation therapists create goals for mobility, transfers, and self-care. ● Social worker creates goals for caregiver support, follow-up care, and discharge disposition.	2–17
	**Meet to plan the patient’s transition to home-based care** (30 min) In dialog with the patient/caregiver, the treating nurse, social worker, and therapists will develop a plan for home-based care, targeting 6 key care needs. ● Nurses focus on medications, advance care planning and symptom management. ● All staff help the patient and caregiver describe their needs for continuing care at home. ● Social worker reviews Transition Plan of Care and the Connect-Home Activation Visit.	5–10
	**Prepare the patient and caregiver for home-based care** (2.5 h) 1. Teach skills and plans for home-based care, targeting 6 key care needs. ● Nurses teach symptom management (e.g., pain), clarify advance care planning preferences, and reconcile medication orders. ● Rehabilitation Therapists teach skills for function and safety at home. ● Social worker schedules and explains appointments, home-based care, and cost. 2. Initiate hand-off to home-based care (over the last 1–2 days before discharge) ● **SNF staff** send medical records and copies of any advance care planning documents to the patient’s physician and the Connect-Home Activation RN. ● **Nurses**: (a) reconcile medications, (b) provide supplies and medications, and (c) re-teach the written Transition Plan of Care and medication list.	6–20
**Step 2.** Transitional care in the patient’s home	**Implement the Transition Plan of Care at home (2 h)** Connect-Home Activation Nurse visits the patient and caregiver at home to: ● Reconcile medications on the discharge medication list and in the home, ● Help family implement new care routines, addressing 6 key care needs, ● Conduct a brief home safety and falls prevention screen, ● Coordinate care with follow-up clinicians and home health care nurses, when applicable.	21

*COVID Modification*. At the end of period 12 of this 22-period study (Fig. 1), the study team was no longer permitted to enter the SNFs; moreover, caregivers of SNF patients were no longer permitted to enter the SNFs. The Connect-Home intervention was modified to address these COVID-related changes in the SNFs. First, in-person staff training was changed to Zoom- and telephone-based staff training. Second, in-person transitional care services with caregivers of SNF patients were changed to Zoom- and telephone-based care. In Connect-Home Step 1, staff members were trained to discuss patient needs and goals by telephone (as opposed to in-person). In Step 2, staff were trained to host care plan meetings by telephone or via Zoom (as opposed to in-person). In Step 3, staff were trained to engage and educate caregivers by telephone or via Zoom. Also, in Step 3, on the day of discharge to home, staff members were trained to discuss final discharge plans by telephone, via Zoom, or in-person in an area outside of the SNF as needed. All other elements of the protocol were not changed.

#### Strategies to improve adherence to Connect-Home interventions

The National Institutes of Health Behavior Change Consortium’s “Treatment Fidelity Protocols” will be used to enhance fidelity to the intervention [44] starting with standardized tools (e.g., Connect-Home Toolkit, the EHR template and staff training protocols) to deliver the intervention. To facilitate fidelity to the training protocol, we will train staff in each SNF and partnering home health care RNs to prevent contamination between SNFs. We will use a detailed training protocol (Table 1) specifying the roles, content, participants, and time required for training activities. To ensure fidelity to delivery of the training protocol, a member of the research team will observe staff training activities on a random schedule to assess fidelity of the trainer to the staff training protocols. The research team will address deviations from the training protocol. We will use a Training Contact Database, in which a research team member will record staff participation in training activities. Staff members will be assigned identification numbers for tracking participation. To ensure receipt of treatment, a research team member will administer post-tests to all participating staff after the staff training session; additional one-to-one re-training will be provided as needed for those who score < 100%. The research team will also use return demonstration of home visit procedures with Connect-Home Activation RNs, followed by re-training as needed until nurses demonstrate 100% ability. To ensure enactment of skills in the intervention protocol, the RC will audit medical records for intervention patients to assess fidelity to the study protocol: (a) completing the Transition Plan of Care; (b) convening care plan meetings; (c) reviewing advance directives in the SNF; (d) scheduling follow-up medical appointments; (e) transmitting records to follow-up clinicians; (f) completing home visits within 24 h after discharge; (g) reconciling medications in the patient’s home; (h) completing the home safety evaluation; (i) communicating patient status to the home health nurse. As part of enactment monitoring, the Connect-Home Activation RN will keep a log describing medication discrepancies and the patients referred for a rehabilitation therapy after discharge home. Further, the research team member will host a 30-min monthly meeting during the intervention phase in each SNF to relay feedback and discuss findings from fidelity monitoring with SNF staff and the Connect-Home Activation RN. Finally, we will observe 10% of staff as they deliver Connect-Home, using a standard checklist to provide feedback about enactment of the Connect-Home protocols; SNFs failing to achieve 70% of operationalized fidelity steps for five or more patients will undergo re-training.

### Outcomes

#### Outcome measures

We will use patient- and caregiver-reported outcome measures to assess intervention effects at 7, 30, and 60 days after SNF discharge (Table 3). The primary outcomes are the patient’s Preparedness for Discharge (CTM-15, range 0 to 100) [36] and Preparedness for Caregiving (summarized as a mean score of eight items, each 0 to 4) [37]; these measures of self-reported readiness to continue healthcare at home will be assessed on post-discharge day 7 when patients and caregivers will have completed the intervention, and patients have adjusted to being home. Secondary outcomes are quality of life, patient function, days of acute care use, and caregiver burden and distress at 30 and 60 days after discharge.

**Table 3:** Concepts measured, measures, data source, and timing (days)

Concept	Measure (with scoring and psychometrics)	Who	7	30	60
**Aim 1**					
Preparedness for discharge	Care Transitions Measure-15 (CTM-15). High reliability (alpha range = 0.93–0.95), 15 items on a 4-point scale, measuring self-reported knowledge and skills for continuing care at home. Standardized summary score range 0–100; higher scores associated with less acute care use after discharge [45, 46].	Pt	x		
Preparedness for caregiving	Preparedness for Caregiving Scale (PCS). Moderate to high reliability (alpha = 0.86–0.92, 8 items on a 5-point Likert scale (0–4), measuring self-reported readiness for caregiving. Range = 0–32; higher scores associated with less anxiety [47–49].	CG	x		
**Aim 2**					
Quality of life	McGill Quality of Life Questionnaire (MQoL). Moderate reliability (alpha = .80), 16 items on a 7-point Likert scale; the scale is recommended for studies of palliative care and measures quality of life across disease trajectories [50, 51].	Pt		x	x
Function	Life Space Assessment. High reliability, (alpha = 0.96), 5 Likert scales corresponding to a hierarchy of levels of mobility (each scored from 0 to 4) where weights are the product of the “Life-space level” (range 1–5) and the “independence” score (range 1–2); range = 1–120. Lower scores are associated with falls and hospitalization [52–55].			x	x
Caregiver burden	Zarit Caregiver Burden Scale. High reliability, (alpha = 0.89), with 12 items on a 5-point scale, measuring caregiver perceptions that “caregiving has an adverse effect on their emotional, social, financial, physical and spiritual functioning.” Scores range 0–48; higher scores associated with depression and social isolation [56, 57].	CG		x	x
Caregiver distress	Distress Thermometer includes 1 item on an 11-point scale, measuring negative affect (e.g., sadness and fear) related to caregiving for a severely ill person. Score ranges 0–10, with scores > 4 associated with poor coping and depression [58].			x	x
**Aim 3**					
Days of acute care use	Self-reported number of combined number of days the patient spends in the ED or hospital in 30 and 60 days after SNF discharge [59].	Pt		x	x
**Exploratory outcomes**					
Falls	Self-reported number of patient falls with injury (those requiring medical attention) and without injury; falls are defined as an “unintentional change in position resulting in a resident coming to rest on the ground or lower level” [60].	Pt		x	x
Hospice enrollment	Self-reported enrollment in hospice (yes/no) in 30 and 60 days after SNF discharge.			x	x
Home health care use	Number of days of home health care provided by nurses and rehabilitation therapists in 30 and 60 days after SNF discharge			x	x
Hospital use	Self-reported count of hospital readmissions, either acute or observational stays after SNF discharge in 30 and 60 days after SNF discharge.			x	x
ED use	Self-reported count of emergency department visits without hospital stay, in 30 and 60 days after SNF discharge.			x	x
Death	Caregiver-reported death of the patient in 30 and 60 days after SNF discharge.	CG		x	x

In addition, we will collect data on covariates about patients from chart reviews and in-person surveys. We will collect data about caregivers in person or by phone. Patient data collected in person will include as follows: frailty (Study of Osteopathic Fractures Index) [61] and social support ENRICHD Social Support Inventory (ESSI) [62]. Patient data collected with SNF chart abstraction will include age, sex, race, ethnicity, health insurance status (Medicare/Medicaid Advantage/Medicare fee-for-service/private), living arrangements before index hospitalization, educational attainment, medical history (primary diagnosis in the hospital discharge summary, hospital care (critical care, surgery and length of stay), depression (Minimum Data Set section D) [39], function (Minimum Data Set section GG) [39], cognitive status (Minimum Data Set section C) [39], SNF care (i.e., SNF length of stay, urgent or acute treatment while in the SNF), and discharge destination. Using the problem list in the medical record, we also will calculate the Charlson Comorbidity Index scores for each patient [63]. Patient data collected with Home Health Care chart abstraction will include number of days of home health care use in 30 and 60 days after SNF discharge (Table 3). Caregiver data collected in person or by phone will include age, sex, relationship to patient, living arrangements, education, employment, and count of days per week providing patient care.

#### Participant timeline

**Fig. 3:**
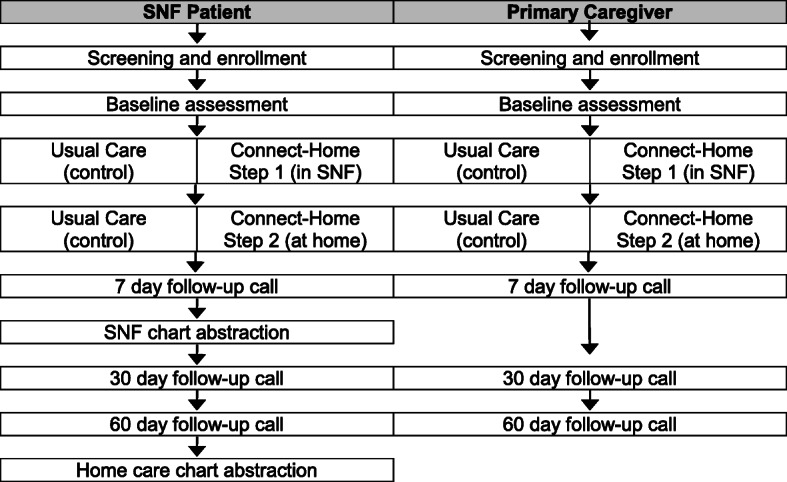
Participant timeline

Patient and caregiver participation in the Connect-Home trial are described in Fig. 3.

#### Assignment of interventions: allocation

The lead study statistician (J.P.) not involved in the day to day conduct of the trial, employed stratified randomization via SAS Proc Plan to randomly assign the six SNFs to treatment sequence or, equivalently, timing of intervention rollout (Fig. 1). SNFs were pair-matched according to perceived readiness to adopt organizational change, i.e., the Connect-Home intervention. The “most ready” pair was randomly assigned to the first and six sequences, essentially based upon a coin toss. The “least ready” pair was similarly assigned to the third and fourth sequences and the remaining pair to the second and fifth sequences, with the final result being one SNF assigned to each sequence. The project manager gave the six SNF generic labels, A, B, C, D, E, and F, which the statistician used to generate the assignments. In this way, SNF identities were concealed until treatment sequences were assigned.

#### Data collection and management

Data collection will be identical for intervention and control periods. Professional data collectors from a research survey lab will collect patient and caregiver outcome data by phone from intervention and control patients and caregivers in 7, 30, and 60 days after SNF discharge and will be blinded to study hypothesis and to treatment group assignment. Also, the RC will administer baseline enrollment surveys to collect measures of covariates in person from intervention and control patients in the SNF. The RC will collect data from caregivers in person or by telephone if necessary. If patients are not able to participate in the recruitment or data collection interview, the RC will collect covariate data from the patient’s caregiver who is a legally authorized representative. Two weeks after patients’ discharge from the SNF, the RC will use a standardized chart abstraction tool to collect additional data from patients’ medical record in the SNF (see Measures of Covariates, below Table 3). The RC will use the same procedure to abstract data from patients’ home health care records in 60 days after SNF discharge. SNF and home visit staff will not be involved in data collection.

#### Plans to promote participant retention and complete follow-up

We will use several retention strategies demonstrated to be successful in our preliminary studies. First, during recruitment, the RC will provide the patient and caregiver with study contact information, a schedule of follow-up calls, and inform them of compensation for completing data collection. Second, the RC will send a reminder letter to patients and caregivers a week before the data collection call. Third, we will compensate participants $120 for post-discharge data collection calls, $60 per patient and $60 per caregiver. We have adjusted our sample size anticipating a 30-day attrition/death rate of 23% based on our pilot studies.

### Statistical methods

We will use an intent-to-treat analysis in which all patients/caregivers who are discharged to home will be included in the analysis according to the randomized treatment allocation of their SNF at enrollment regardless of whether they receive the full intervention or whether the intervention was rolled out in the SNF exactly on the first Monday of the assigned month. Some patients will transfer from the SNF to the hospital, to long-term care, or will die in the SNF and therefore will not discharge from the SNF to home. Although these patients (and their caregivers) have initially enrolled, they cannot provide outcome measures and will not be included in the analysis. Otherwise, all available data on patients who are discharged home will be used in analyses through their planned study endpoint at 60 days post-discharge or until they are no longer living at home, i.e., death or hospitalization. For continuous outcome variables that are highly skewed, we will log-transform the data for analysis. All statistical tests will be two-tailed with 0.05 significance levels. Finally, in the absence of strong temporal effects, we expect balance in covariates between control and intervention patients; supplemental analyses will involve assessing the impact of the covariates (i.e., insurance provider, SNF length of stay, race, gender, and others) on outcomes in the statistical models for Aims 1–3. Covariates will be imputed with the mode of imputation based upon the degree of missingness; multiple imputation will be used if more than 15% of study participants have missing data [64]. Missing outcomes will not be imputed.

*COVID modification*. At study pause, we conducted a post hoc assessment of covariate imbalance between treatments conditions with respect to age, gender, and race of residents. After 12 study months, the proportion of enrolled male residents was statistically significantly higher (*p* < 0.05) in the intervention than in the control condition. While gender balance would ordinarily be expected using our recruitment procedures, such might not be the case in the stepped wedge trial if the proportion of male SNF admissions increases as a result of the COVID-19 pandemic. Therefore, we will monitor recruitment by gender for the duration of the trial and increase efforts to enroll female residents if deemed necessary.

#### Aim 1 analysis

We will use linear mixed models to compare observations between intervention and usual care periods for the study’s two primary outcomes: patient Preparedness for Discharge score (CTM-15) and Preparedness for Caregiving score, both measured at 7 days post-discharge. Linear mixed models allow for different numbers of patients per site (SNF), while accounting for correlated responses between patients within the same SNF. Let *Y*_*ijk*_ denote the outcome for patient *k* in SNF *i* and period (enrollment month) *j*; (*i* = 1, …,6; *j* = 1, …,22). The primary analysis model is

$$ {Y}_{ij k}={\beta}_0+{\alpha}_i+{\beta}_1j+{\gamma}_{j(i)}+\theta {X}_{ij}+{e}_{ij k}, $$

where *β*_0_ is an intercept term, *α*_*i*_ is a random effect for SNF, *β*_1_ accounts for a linear temporal trend, *γ*_*j*(*i*)_ is a random effect for period j nested within SNF *i*, *X*_*ij*_ is an indicator for the treatment condition (i.e., 1 if Connect-Home; 0 is standard discharge procedures) during period *j*, *θ* is the treatment effect, and *e*_*ijk*_ is an error term. Furthermore, we assume a variance components structure such that all random effects are independent with ${\alpha}_i\sim N\left(0,{\sigma}_{\mathrm{c}}^2\right)$, ${\gamma}_{j(i)}\sim N\left(0,{\sigma}_{\mathrm{p}}^2\right)$ and ${e}_{ijk}\sim N\left(0,{\sigma}_{\mathrm{e}}^2\right)$ where subscripts “c,” “p,” and “e” denote cluster (i.e., SNF), period and error variance components, respectively. The nested exchangeable correlation structure induced by these random effects allows the within-SNF correlation of outcomes from two patients enrolled in the same month (ICC_W_) to be different from the within-SNF correlation of outcomes from patients enrolled in different months (ICC_B_) [65, 66]. Specifically, by denoting the total variance ${\sigma}_T^2={\sigma}_c^2+{\sigma}_p^2+{\sigma}_e^2$, the intraclass correlations are ICC_W_ = $\left({\sigma}_c^2+{\sigma}_p^2\right)/{\sigma}_T^2$, and ICC_B_ = ${\sigma}_c^2/{\sigma}_T^2$. The group difference in outcome scores (intervention vs. control) across the study periods will be estimated along with a 95% confidence interval. In the model for patient preparedness for discharge (CTM-15 score), we will include data provided by the patient or by caregiver as a proxy (for patients who are not able to answer survey questions). Due to skewness of the CTM-15 score distribution found in our preliminary data, we anticipate that this outcome will be log-transformed prior to analysis. In addition to the main analysis for each primary outcome, two modifications of the model will be fitted as sensitivity analyses. First, the primary model will be amended by including additional explanatory variables as fixed effects: patient age, gender, race, and for CTM-15 score only, source (patient or caregiver). Second, we will conduct a sensitivity analysis to assess whether the effect of Connect-Home depends on the number of months that an SNF has been operating under the Connect-Home protocol. This is achieved by extending the linear mixed model so that separate effects of Connect-Home are estimated when the Connect-Home protocol has been implemented for ≤ 3 months versus > 3 months in order to examine whether the impact of Connect-Home is sustained 3 months following its initial implementation in the staff training period.

#### Aim 2 analysis

In Aim 2, we will use linear mixed models that are extensions of those in the Aim 1 analysis for the analysis of secondary outcomes: quality of life (MQoL), patient function (Life Space Assessment), caregiver burden (Zarit scale), and caregiver distress (Distress Thermometer). We will augment the linear mixed model equation from Aim 1 with a random effect for patients and additional fixed effects for visit and treatment by visit interactions to model the day 30 and 60 outcomes simultaneously as repeated measures. A statistically significant visit by treatment condition interaction would indicate that the impact of Connect-Home varies according to elapsed days since discharge. Because our main interest is in day 30 and 60 outcomes, we will examine for differential treatment effects at these two time points and, if differences exist, estimate the effect of the Connect-Home intervention and its 95% confidence at each time point.

Next, for the exploratory outcome number of falls in hypothesis 2a, we will use Poisson mixed models with log link to assess the intervention effect on falls with a similar structure for fixed and random effects as described in the Aim 1 analysis [67]. Instead of modeling the repeated outcomes as the number of falls between day 0–30 and between days 31–60, respectively, we separately model the cumulative number of falls by day 30 and 60, respectively, to assess whether the Connect-Home intervention reduces the cumulative fall rates post-discharge at these two time points. We will estimate the incident rate ratio and its 95% confidence interval for falls comparing intervention and control conditions.

#### Aim 3 analysis

The main outcome for Aim 3, the number of cumulative days of acute care use (i.e., days due to rehospitalizations and emergency department visits) will be modeled using separate marginalized zero-inflated Poisson (MZIP) random intercept models with log link for 30 and 60 days after discharge, respectively [67]. We will test and estimate the overall effect of Connect-Home relative to standard discharge procedures through exponentiation of the treatment regression coefficient for the overall mean as an incidence rate ratio (IRR) and its 95% confidence interval, employing empirical standard errors to allow for possible extra-Poisson variation. We chose marginalized zero-inflated Poisson (MZIP) over the standard zero-inflated Poisson (ZIP) model because although a large number of zero counts (event outcomes) are anticipated, the overall exposure effect of Connect-Home is of interest, and not its effect on an unobserved subpopulation (i.e., latent class of “at-risk” patients). Next, we will use separate time point-specific generalized linear mixed models (GLMM) with logit link for the binary outcome of whether a resident visited the ED within 30 or 60 days of discharge, respectively, from the SNF. This model type is an extension of ordinary logistic regression that includes random intercepts and period effects for SNFs to account for the within-SNF correlation induced by the stepped wedge design. We will apply a similar GLMM to compare the proportion of hospice referrals between standard discharge procedures and Connect-Home. When events are sparse, we will treat SNFs as fixed effects to mitigate model non-convergence problems.

#### Exploratory aim analysis

To determine the extent to which the intervention effect can be attributed to home health care use, we will use a general multilevel approach to causal mediation analysis to evaluate home health care use as a mediator for intervention effect on two secondary outcomes at 30 days follow-up, including count of [68] patient days of acute care use and caregiver burden [69]. This approach uses two random effects models within the counterfactual framework: (a) for the mediator (number of days of home health care use) as a function of treatment condition and covariates and (b) for the outcome as a function of treatment condition, mediator, and covariates. Outputs include indirect and direct effects of the intervention [69].

#### Sample size

We used computer simulations to calculate statistical power for comparing control (standard discharge procedures) and intervention (Connect-Home) conditions for our primary outcomes [(Preparedness for Discharge (CTM-15 score) and Preparedness for Caregiving], and selected secondary outcomes. For each scenario, we generated 1000 simulated datasets with outcomes clustered within SNFs for the stepped wedge design in Fig. 1 and the respective linear mixed models (linear mixed model equation defined above for Log CTM-15, Preparedness for Caregiving Scale, MQoL, Life Space Assessment, Zarit Caregiver Burden scale) and MZIP random intercept model (days of acute care use). Based on preliminary data for Aim 1, we assumed that the group difference in log CTM-15 scores was *θ* = 0.15. We also assumed that responses from patients within the same SNF in the same period would have an intra-cluster correlation of 0.10 (ICC_W_) and that responses from patients or caregivers in the same site but from different periods would have an intra-cluster correlation of 0.05 (ICC_B_); together with the total variance of the outcome (SD^2^), these values determine ${\sigma}_c^2,{\sigma}_p^2$, and ${\sigma}_e^2$ in the linear mixed models of Aims 1 and 2. Under these assumptions and accounting for 23% dropouts, 277 patients at 7 days post-discharge will provide 89% power to detect an increase of 0.15 in the mean log CTM-15 score (equivalent to a 16% increase in the mean CTM-15 score) among intervention patients relative to control patients, using a two-sided test at the 5% significance level (Table 4); see “Trial Status” section below for power updates). Power for Caregiver Preparedness is similarly determined but without the logarithmic transform. Power for the Aim 2 secondary outcomes at 30 days post-discharge assumes a 30% dropout rate. For days of acute care use in Aim 3, we assumed a control group prevalence of 22% of patients having at least one day of acute care use and, among those patients, a truncated-at-zero mean of 4.0 days. These assumptions imply that the overall mean number of acute care use days is 0.881 and the excess zero probability is 0.776. For the intervention group, we assumed an overall mean of acute care use days of 1.8 and excess zero probability 0.685. Thus, assuming 30% dropout and (optimistically) zero intraclass correlation within SNFs, the MZIP model with empirical standard errors adjusting for possible clustering within SNFs has 71% power (Table 4). If there is positive intraclass correlation, power will be lower. However, for days of acute care use within 60 days of discharge, power is higher than for 30 days due to higher prevalence and mean number of days of acute care use.

**Table 4:** Power to detect intervention effects for measures with 360 patients enrolled

Aim	Outcome^a^	Effect Θ	S.D.	Power (%)		
				Percent dropout^b^		
				0%	23%	30%
1	Patient preparedness (Log CTM-15)	0.15	0.046	99	89	82
1	Preparedness for caregiving	0.5	0.77	84	78	75
2	Quality of life (MQOL)	1.0	1.5	86	80	77
2	Function (Life-Space)	17.5	25	98	88	83
2	Caregiver burden	6.0	9	96	86	80
3	Days of acute care use^c^	0.5	NA	82	74	71

^a^Log CTM-15 and caregiver preparedness are 7 days post-discharge; others are 30 days post-discharge. ^b^We anticipate 277 patients with 7 days post-discharge outcomes (23% dropout) and 256 patients at 30 days (30% dropout). ^c^For Aim 3, *θ* is the incidence rate ratio

## Discussion

This study is designed to evaluate whether Connect-Home improves transitions of care for SNF patients being discharged home and their caregivers. The study is designed to be the first adequately powered efficacy trial of transitional care services designed for seriously ill SNF patients and their caregivers. The Connect-Home intervention focuses on the key care needs of aged SNF patients and their caregivers, such as advance care planning and symptom management. It will also focus on the patient and caregiver as a team, rather than the patient alone. Connect-Home is innovative by embedding new tools in the EHR and related organizational structural changes to SNF care delivery. Finally, Connect-Home is innovative in its reliance on existing SNF staff and home health RNs to deliver transitional care, which would enhance its implementation in real-world settings should it demonstrate effectiveness.

Findings from the study should be considered in light of potential problems and challenges in the way it was designed. First, the study will include SNF patients with diverse medical conditions. One approach to sampling to minimize outcome variation due to underlying disease heterogeneity (and therefore type 2 error) would be to restrict to a single admitting diagnosis. However, SNF patients typically have two or more serious medical conditions and functional impairment. Thus, generalizable transitional care tools and training are needed for staff caring for all seriously ill patients who transfer home, not only those with specific disease states [13, 18, 24, 70]. Moreover, sampling based on one or a few disease states would reduce the feasibility of achieving the sample size for the proposed efficacy trial. To address the potential for type 2 error due to variation in outcome measures, we have (a) powered the sample size using outcome variance from a robust pilot study in a heterogeneous population [33]; (b) measured the primary outcome with the CTM-15, which is less sensitive to patient heterogeneity than rehospitalization [36]; and (c) used a stepped wedge design to incorporate both across and within-facility changes in outcomes assessment considering that case-mix is likely to remain more stable within facilities than between facilities.

The Connect-Home study will provide evidence to guide care for seriously ill older adults and their caregivers who are rarely the focus of intervention research. In the USA, alone, results of the study will inform care of more than one million older adults each year. Findings will be among the first to described unique transitional care services designed for SNF patients and for their caregivers. Moreover, Connect-Home has the potential to generate a new model of transitional care not dependent on research staff for delivery; thereby, accelerating translation of the intervention from research to clinical practice. The findings from this study also will inform future implementation research and testing in a large, pragmatic trial. For example, the findings will include methodology for embedding the Transition Plan of Care EHR template to create individualized plans prior to the transition from SNF to home-based care. Findings will also include an approach for implementing Connect-Home, using fidelity measurement and strategies to provide direct feedback to clinical staff, that can be applied in a large-scale effectiveness study.

### Trial status

At the time this report was submitted for publication, the intervention was being delivered to patients and caregivers in three of six SNFs through twelve completed periods/months of the study design (Fig. 1). The study protocol (NCT03810534), version number one, was registered on January 18, 2019. Recruitment began in March of 2019 and was expected to be completed in approximately March 2021. In response to the COVID-19 pandemic, recruitment was paused on March 7, 2020, and restarted—after the original submission of this publication—on August 10, 2020. At the time of the pause, 212 SNF patients and 212 caregivers had been enrolled. During the pause, as described above, we modified the protocol to mitigate the risks of COVID infection. These modifications (risk mitigation strategies) were IRB approved and registered in ClinicalTrials.gov. It is important to note that the COVID-19 pandemic temporarily affected the ability of the SNF staff and the study team to undertake any research activities, which included screening and recruiting patients and caregivers. Thus, COVID affected the design of the study by forcing a pause early in period/month 13, thereby altering the SNFs’ beginning and end dates for three phases of patient and caregiver enrollment. These COVID-related changes in our design did not affect the intention-to-treat analysis, in which all patients/caregivers who are discharged to home will be included in the analysis according to the randomized treatment allocation of their SNF at enrollment regardless of whether they receive the full intervention or the actual date the intervention was rolled out in each SNF. Moreover, the primary intention-to-treat analysis based on the a priori-specified linear mixed model described above will not be adjusted. At this time, it is unknown how the COVID-19 pandemic affects the older adults and caregivers who receive care in SNFs. Future exploratory analyses of the study’s primary outcomes will include an adjustment to the assumed linear time trend in the linear mixed model, based on the timing of the study pause to adjust for the impact of COVID-19 on outcomes among the study participants.

At the study pause, we updated the original power analysis based upon a post hoc interim statistical analysis that included estimation of temporal trends and variance components for the two primary outcomes in linear mixed models that did not include the treatment indicator, thus maintaining the nominal type I error rate in final outcome analyses. The interim analysis found that the distribution of CTM-15 is approximately normally distributed and does not require logarithmic transformation; therefore, both updated power analysis and final statistical analysis will be based on untransformed CTM-15. In the interim analysis of residents with day 7 follow-up CTM-15 outcomes (*n*=160), the standard deviation (SD) of 17.7 was about 25% larger than in the pilot data, whereas the ICC was essentially zero and inestimable; therefore, we conservatively set ICC_W_ = ICC_B_ = 0.005 in the updated power simulations. The increased magnitude of SD had the largest influence such that simulated power based on *n* = 277 participants (allowing for 23% dropout) was 74% to detect at study closeout a difference in CTM-15 between treatments of 10.0, and 80% power to detect a difference of 12.0 for an 18% increase in CTM-15 due to the intervention (e.g., an increase from a mean CTM-15 score of 65 to 77 instead of 65 to 75 in the pilot data). Next, for Preparedness for Caregiver, the interim analysis (*n* = 158) found that SD was very similar as in the pilot data (Table 4) and thus it was not altered in the simulations. However, interim power was slightly lower than expected because the within-period ICC was larger (ICC_W_ = 0.184) in the interim analysis, compared to the value originally assumed. In particular, assuming updated values ICC_W_ = 0.184 and ICC_B_ = 0.037, *n* = 277 (allowing for 23% dropout) provides 67% power to detect a difference in Preparedness for Caregiver mean score of 0.5, and 82% to detect a difference of 0.6. Finally, the interim simulations established that the nominal type I error rate of 0.05 for tests of intervention was maintained with use of the Kenward-Roger (KR) finite-sample bias correction (given Connect-Home is in 6 SNFs); therefore, while not originally planned, final analyses will use the KR correction in all linear mixed models analyses. In summary, the interim power analysis, updated according to estimated temporal effects, SDs and ICCs, shows that the planned, a priori statistical analysis provides approximately 80% power to detect differences with two-sided *α*-0.05 tests of 12.0 and 0.6 for CTM-15 and Preparedness for Caregivers, respectively, which differs from the minimal detectable effects sizes of 10.0 and 0.5, respectively, in the original power analyses. The updated power analysis does not account for potential unknown effects of COVID that may dampen the effect of intervention suggesting that the Connect-Home trial could possible achieve clinically important effects that are not statistically significant. Therefore, results will be reported with 95% confidence intervals for estimated effects with full transparency for pre-planned analyses and designation of outcomes and analyses as primary, secondary, and exploratory/sensitivity as detailed in the protocol presented in this article.

## Data Availability

Reasonable requests will be will be considered by the authors with additional permission of the Institutional Review Board at the University of North Carolina at Chapel Hill.

## Abbreviations

US: United States
SNF: Skilled nursing facility
ED: Emergency department
EHR: Electronic health record
CTM-15: Care Transitions Measure-15
RN: Registered nurse
LPN: Licensed practical nurse
DON: Director of nursing
MDS: Minimum Data Set Nurse
NP: Nurse practitioner
PT: Physical therapist
OT: Occupational therapist
ST: Speech therapist
COTA: Certified occupational therapy assistant
PTA: Physical therapy assistant
RC: Recruitment coordinator
IRB: Institutional review board
ESSI: ENRICHD Social Support Inventory
MQoL: McGill Quality of Life Inventory
SD: Standard deviation
ICC: Intraclass correlation coefficient
MZIP: Marginalized zero-inflated Poisson
ZIP: Zero-inflated Poisson
GLMM: Generalized linear mixed models
PI: Principal investigator
AE: Adverse event
SAE: Serious adverse event
SMC: Safety Monitoring Committee
